# Combination of H1 and H2 Histamine Receptor Antagonists: Current Knowledge and Perspectives of a Classic Treatment Strategy

**DOI:** 10.3390/life14020164

**Published:** 2024-01-23

**Authors:** Erwen Kou, Xiaobei Zhang, Baiping Dong, Bo Wang, Yuanjie Zhu

**Affiliations:** 1 Department of Dermatology, Naval Medical Center, Naval Medical University, Shanghai 200052, China; kouew@smmu.edu.cn (E.K.); dbpjuly@smmu.edu.cn (B.D.); 2 Department of Pharmacy, Naval Medical Center, Naval Medical University, Shanghai 200052, China; zhaohaixia@smmu.edu.cn

**Keywords:** histamine, histamine receptor antagonists, combination therapy, H1 receptor, H2 receptor, histamine-mediated diseases, clinical application

## Abstract

Histamine receptor antagonists, which can bind to specific histamine receptors on target cells, exhibit substantial therapeutic efficacy in managing a variety of histamine-mediated disorders. Notably, histamine H1 and H2 receptor antagonists have been extensively investigated and universally acknowledged as recommended treatment agents for numerous allergic diseases and acid-related disorders, respectively. Historically, the combination of H1 and H2 receptor antagonists has been considered a classic treatment strategy, demonstrating relatively superior efficacy compared with single-drug therapies in the treatment of diverse histamine-mediated diseases. The latest emerging studies have additionally suggested the underlying roles of histamine and H1R and H2R in the development of anxiety disorders, arthritic diseases, and postexercise hypotension. Nevertheless, there is still a lack of systematic reviews on the clinical efficacy of combination therapy, greatly limiting our understanding of its clinical application. Here, we present a comprehensive overview of the current knowledge and perspectives regarding the combination of H1 and H2 histamine receptor antagonists in various histamine-mediated disorders. Furthermore, we critically analyze the adverse effects and limitations associated with combination therapy while suggesting potential solutions. Our review can offer a systematic summary and promising insights into the in-depth and effective application of the combination of H1 and H2 receptor antagonists.

## 1. Introduction

**Figure 1:**
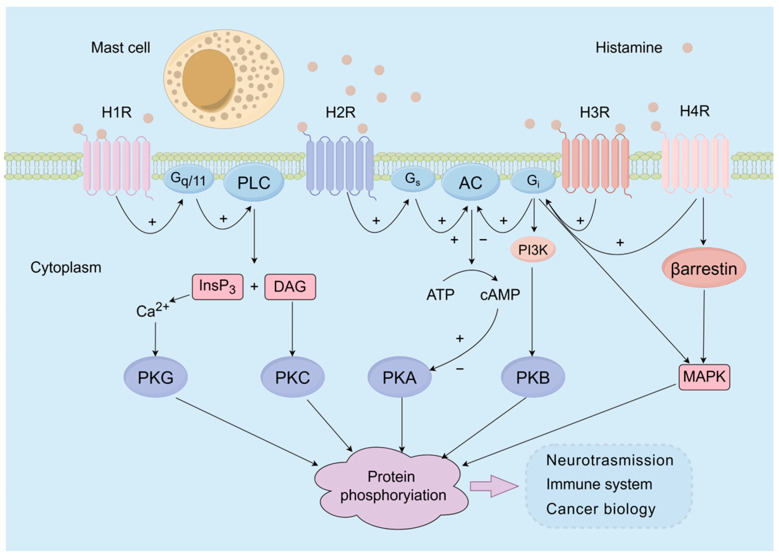
Schematic overview of the main signaling pathways by which histamine exerts its biological effects on target cells. Presently, four subtypes of histamine receptors have been identified, namely, H1R, H2R, H3R, and H4R. H1R exerts its effects mainly by coupling to Gq/11 proteins and subsequently elicits the activation of phospholipase C. This lipase can produce 1,2-diacylglycerol and inositol-1,4,5-trisphosphate, leading to the activation of protein kinase C (PKC) and the release of intracellular calcium ions, respectively. H2R can stimulate the production of cAMP-PKA by coupling to Gs proteins. Gi can be activated via H3R or H4R and subsequently activate cAMP and PI3K, leading to the subsequent activation of PKA and PKB. In addition, the activation of Gi proteins by H3R and H4R results in the activation of mitogen-activated protein kinase (MAPK) pathways.

Histamine, an endogenous biogenic amine, is predominantly localized within the cytoplasmic granules of mast cells and basophils, and its release is elicited by a diverse array of immune and nonimmune stimuli [1]. Histamine exerts significant regulatory effects on numerous physiological and pathological processes, including neurotransmission, immune function regulation, capillary dilation, cytokine production, and gastric acid secretion, through its interaction with distinct subtypes of histamine receptors (Figure 1). Presently, four subtypes of histamine receptors have been identified. The histamine 1 receptor (H1R) is widely distributed in various body tissues, including the endothelium, immune cells, and smooth muscle, and plays a crucial role in regulating vasodilation, atrial muscle contractility, and bronchoconstriction [2]. The histamine 2 receptor (H2R) is mainly associated with the cAMP system and primarily governs gastric acid secretion, a process closely linked to the development of peptic ulcers [3]. Furthermore, the histamine 3 receptor (H3R) is distributed predominantly in the nervous system and functions as a presynaptic self-receptor. Finally, the histamine 4 receptor (H4R) is the most recently identified histamine receptor. This receptor shares similar biological and pharmacological characteristics with H3R and is primarily expressed on cells involved in inflammation and the immune response [4]. H4R exerts effects on chemotaxis as well as cytokine and chemokine production in mast cells, eosinophils, dendritic cells, and T cells [1].

Histamine receptor antagonists, which can selectively bind to specific histamine receptors on target cells and block the action of histamine, exhibit notable therapeutic efficacy in managing a variety of histamine-mediated disorders [5]. Currently, the most clinically significant applications of histamine receptor antagonists involve their interaction with H1 or H2 receptors [6]. Notably, second-generation H1 receptor antagonists, such as loratadine and cetirizine, are recommended as standard treatments for several allergic diseases, such as urticaria [7], allergic rhinitis [8], and atopic dermatitis [9]. Meanwhile, H2 receptor antagonists have been recognized as the standard treatment guidelines for several acid-related disorders, such as gastric and duodenal ulcers and esophagitis [10]. The H1R and H2R antagonists that are currently clinically used are listed in Table 1.

**Figure 2:**
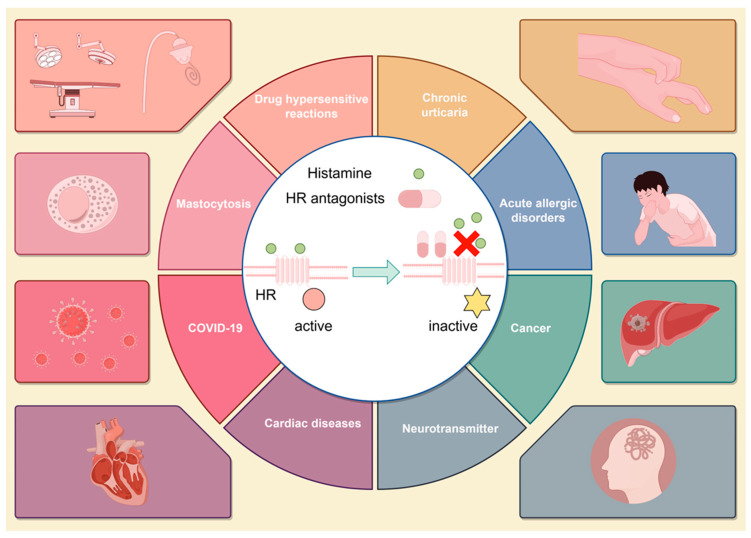
The combination of H1 and H2 receptor antagonists as a classic or potential treatment for various diseases.

Historically, the combination of H1 and H2 receptor antagonists has been regarded as a classic treatment strategy [29]. Due to the role of histamine in a variety of diseases, particularly the joint participation of H1R and H2R in the majority of pathological processes, the combination of H1 and H2 receptor antagonists has been gradually investigated in the treatment of diverse histamine-mediated disorders, such as urticaria [30], mastocytosis [31], and other hypersensitive reactions [32], exhibiting a relatively superior efficacy compared with single-drug therapies. In addition, the latest emerging studies have suggested the underlying roles of histamine and H1R and H2R in the development of central nervous system disorders [33], arthritic diseases [34], and postexercise hypotension [25]. Thus, a combination of H1 and H2 receptor antagonists may also exhibit potential therapeutic effects on these histamine-mediated disorders (Figure 2).

In this review, we provide a comprehensive summary of the current understanding and application of combination therapy involving H1 and H2 receptor antagonists for the treatment of histamine-mediated disorders. By providing an extensive overview, we strive to offer promising perspectives for the effective and in-depth utilization of this classic combination therapy.

## 2. Methodology

The literature search was conducted using various databases, including PubMed, Web of Science, ScienceDirect, Google Scholar, and clinicaltrials.gov accessed on 15 October 2023. No limitations were implemented on the publication dates of the articles, and the search encompassed various article types, such as books and documents, clinical trials, meta-analyses, randomized controlled trials, reviews, and systematic reviews. “H1R and H2R antagonists” and “combination therapy” were the search terms used. The search strategy was designed to precisely find the relevant literature addressing the therapeutic application and potential effect of the combination of H1R and H2R antagonists in diverse histamine-mediated disorders.

Additionally, all titles and abstracts we cited were independently screened. Articles for extensive and detailed review included those studies that were conducted on human subjects and available in full text, which ensured the integrity and reliability of the data stated in the manuscript. Lastly, studies from the literature included in our review were limited to those published in the English language.

## 3. The Combination of H1 and H2 Receptor Antagonists as a Classical Treatment Strategy

The combination of H1 and H2 receptor antagonists is considered a classic treatment strategy and has demonstrated superior efficacy and safety compared with single-drug therapies for the treatment of diverse histamine-mediated disorders.

### 3.1. Acute Allergic Disorders

Acute allergic disorders are mediated by the release of various chemical mediators and are manifested by a spectrum of clinical symptoms, including dizziness, skin flushing, rash, and dyspnea [35]. Antihistamine therapy is considered a crucial treatment for acute allergic syndromes, and H1 receptor antagonists have exhibited relative safety and efficacy in treating patients with nonthreatening acute allergic syndromes [36]. However, certain patients may experience more severe allergic reactions or experience recurrent manifestations even after receiving initial treatment with H1 receptor antagonists [37]. Therefore, it is particularly necessary to combine H1 receptor antagonists with H2 receptor antagonists for these patients. Moreover, their safety and efficacy have been shown to be satisfactory not only in more severe cases but also in enhancing clinical improvement in emergency cases [12].

In a prospective double-blind study [11], the efficacies of cimetidine and diphenhydramine were compared both individually and in combination for the treatment of symptoms associated with acute allergic reactions. A total of 39 patients were enrolled in the study, 14 of whom were randomly assigned to receive 50 mg diphenhydramine along with a placebo, 12 of whom were assigned to receive 300 mg cimetidine along with a placebo, and 13 of whom were assigned to receive both diphenhydramine and cimetidine. The results indicated that diphenhydramine exhibited greater effectiveness than cimetidine, while the combination of diphenhydramine and cimetidine demonstrated superior efficacy compared to diphenhydramine alone in the treatment of acute urticaria symptoms.

In 2000, a large study was also conducted to investigate the efficacy of combination therapy for the treatment of acute allergic disorders [12]. This study involved 91 adult patients with acute allergic syndrome who were randomly assigned to receive either 50 mg diphenhydramine and saline solution or 50 mg diphenhydramine and 50 mg ranitidine. The results revealed that the proportion of patients without urticaria in the diphenhydramine–ranitidine group (91.7%) was significantly greater than that in the diphenhydramine–placebo group (73.8%) at both 1 and 2 hours after treatment.

These studies demonstrated that the combination of H1 and H2 receptor antagonists can improve the clinical outcomes of patients with acute allergic syndromes compared with the use of either H1 or H2 antagonists alone. Consequently, combination therapy involving H1 and H2 receptor antagonists is recommended for the treatment of acute allergic disorders.

### 3.2. Chronic Urticaria

Chronic urticaria (CU) is a mast cell-mediated dermatological disorder characterized by the recurring appearance of wheals accompanied by intense pruritus that lasts for more than six weeks [38]. The prevalence of CU exceeds 2%, and CU imposes significant physical and psychological distress upon affected individuals [39]. The primary therapeutic approach for chronic urticaria involves the administration of H1 receptor antagonists [40]. However, there is significant variability in the effectiveness of H1 receptor antagonists among patients, with approximately half of them experiencing no significant improvement after receiving treatment [41]. Therefore, supplementation with adjuvants, such as H2 receptor antagonists, leukotriene receptor antagonists, antianxiety drugs, and Chinese herbal drugs, is necessary for these patients [42,43].

The combination of H1 and H2 receptor antagonists has been utilized since as early as 1978, when it was reported to be effective for patients with chronic urticaria who were unresponsive to conventional drugs [44]. Similarly, the addition of cimetidine 800 mg/day was effective in very few patients who did not respond to conventional H1 receptor antagonists [45]. In a study on chronic idiopathic urticaria, the administration of chlorpheniramine 5 mg four times daily and cimetidine 400 mg four times daily for a duration of 4 weeks demonstrated certain advantages compared to the use of chlorpheniramine alone [15]. Additionally, a randomized double-blind study involving 45 patients investigated the effects of 60 mg of terfenadine twice daily, 150 mg of ranitidine twice daily, or a combination of both. The results suggested that the combination therapy group experienced the least itching, although there were no discernible differences in the severity of wheals or edema, further verifying the superiority of combination therapy in attenuating the pruritus presenting in chronic urticaria [23].

In Japan, a retrospective cohort study evaluating the clinical efficacy of the addition of lafutidine in the treatment of patients with chronic urticaria who were not controlled with H1 receptor antagonists was performed [28]. Moderate or greater improvement was achieved in 39 of the 46 patients (85%) after 3 weeks of additional administration of lafutidine, and 35 patients (76%) experienced improvement after 3 months. Moreover, no incidence of drug-related adverse reactions was reported. As a representative H2 receptor antagonist, lafutidine appears to be a promising addition to H1 receptor antagonist therapy for the treatment of chronic urticaria resistant to treatment with H1 receptor antagonists alone. The above results fully illustrate the significant advantages of combination therapy for the treatment of chronic urticaria.

### 3.3. Cancers and Carcinoids

Emerging evidence indicates that histamine can significantly promote the development and progression of certain cancers and carcinoids [46]; thus, the use of histamine receptor antagonists may constitute a therapeutic strategy for the treatment of certain cancers and carcinoids. Primary sclerosing cholangitis (PSC) is a chronic inflammatory condition and is considered a prominent risk factor for the development of cholangiocarcinoma (CCA) [47,48]. Recent studies have confirmed that histamine can interact with histamine receptors to promote liver damage and hepatic fibrosis in human PSC and CCA models, mostly through the activation of both IP3/Ca21- and cAMP-dependent signaling mechanisms [49,50,51,52]. Kennedy et al. first examined the efficacy of H1/H2 receptor antagonists on PSC and CCA in mouse models [17]. Wild-type and multidrug-resistant knockout (Mdr^2−/−^) mice were treated with osmotic minipumps containing saline, mepyramine, ranitidine, or a combination of mepyramine and ranitidine for 4 weeks. The results showed that in Mdr^2−/−^ mice treated with H1 and H2 receptor antagonists (alone or in combination), liver and biliary tract injury and fibrosis were significantly attenuated compared with those in Mdr^2-/-^ mice treated with saline, and the treatment of H1 and H2 receptor antagonists (alone or in combination) could reduce tumor growth, serum histamine levels, angiogenesis, and epithelial mesenchymal transition (EMT) volume. This study demonstrated that the application of H1 and H2 receptor antagonists alone or in combination may be therapeutically beneficial for ameliorating both PSC and CCA progression [17].

Additionally, Shi et al. reported that microbiota-produced histamine exerts a bidirectional regulatory effect on the development of gastrointestinal cancer through interactions with the H1 and H2 receptors [53]. A study conducted in rodents further demonstrated that high doses of terfenadine and roxatidine significantly inhibit the growth of tissue chromogranin, histamine, and chromaffin cells in the gastric mucosa of rodents with chronic gastric acid induced hypergastrinemia, thus preventing the formation of carcinoids [24]. Histamine is an intermediate product of the immune response and has complex regulatory effects on the phenotype and function of different immune cells, mainly through the binding to its receptor. Given the impact of histamine on gastrointestinal epithelial and immune cells, simultaneous modulation of the H1R and H2R signaling pathways may be a promising therapeutic approach for the prevention and treatment of inflammation-associated gastrointestinal cancer.

### 3.4. Mastocytosis

Mastocytosis is characterized by the accumulation of a large number of mast cells in various organs and tissues of the body, resulting in a series of pathological changes in tissues and organs. Mastocytosis can be further categorized into cutaneous mastocytosis (CM), systemic mastocytosis (SM), and a rare aggressive form known as mast cell sarcoma (MCS) [54]. Due to the absence of a definitive etiology, the management of mastocytosis primarily focuses on symptom control, including the use of H1 and H2 receptor antagonists and corticosteroids [55].

The administration of either H1 or H2 antagonists alone has demonstrated efficacy in alleviating symptoms, such as cutaneous inflammation and gastrointestinal manifestations arising from mast cell degranulation. However, this approach may not yield satisfactory outcomes in patients with more severe conditions. Notably, the combination of H1 and H2 receptor antagonists might achieve an enhanced symptom control [18]. The combination therapy can inhibit the action of histamine by blocking both H1 and H2 receptors, thereby exhibiting the potential to mitigate the severe symptoms of mastocytosis. It should be noted that the treatment of mast cell syndrome should be individualized according to the specific symptoms and conditions of the patient [56]. When employing this combination therapy, adherence to the physician’s guidance is crucial, and patients should remain vigilant regarding potential adverse effects and drug interactions. Furthermore, alternative treatment strategies, including corticosteroids, adrenaline, and mast cell inhibitors, may be incorporated for individuals with more severe manifestations of the disease [55].

### 3.5. COVID-19

Coronavirus Disease 2019 (COVID-19) is an infectious respiratory disease caused by severe acute respiratory syndrome coronavirus 2 (SARS-CoV-2) that has resulted in a heavy burden on human life and economic development globally [57]. In February 2020, Chinese researchers conducted a comprehensive analysis of all proteins encoded by SARS-CoV-2, followed by the construction of protein and mass structures using homology modeling techniques. Through target-based virtual ligand drug screening, famotidine has emerged as a promising therapeutic option for the treatment of COVID-19 [58]. Furthermore, molecular docking studies have suggested that either of the two SARS-CoV-2 proteases are potential targets of famotidine activity [59].

Subsequently, a series of studies documented the use of famotidine or other histamine receptor antagonists for ameliorating the inflammatory responses and symptoms exhibited by COVID-19 patients [60,61]. Dooley et al. conducted a cohort study examining the effects of cetirizine–famotidine on 110 COVID-19 hospitalized patients with severe pulmonary symptoms. Due to the limitations of not being placebo-controlled, randomized, and blind, and lacking a sufficient number of untreated SOC (standard of care) patients for use as a retrospective control cohort, researchers have compared their findings with cohort data from published studies. The results demonstrated that combination therapy significantly reduced the mortality of hospitalized patients and improved their clinical manifestations compared to patients who were not treated with cetirizine–famotidine or were only treated with famotidine. This illustrated the superior efficacy of the combination of H1 and H2 receptor antagonists [19].

Additionally, a clinical trial involving 214 COVID-19 patients treated with the combination of famotidine and loratadine was completed (NCT05043350). In this trial, the experimental group was treated with standard therapy plus famotidine and loratadine, while the control group was treated with standard therapy plus famotidine alone. The results of this study further verified the superior efficacy and safety of the combination of famotidine and loratadine for the treatment of COVID-19. Notably, a study conducted by Yang et al. reported that the utilization of antihistaminic medications might promote significant immune modulation that helps to manage the cytokine storm caused by COVID-19, and famotidine could attenuate cytokine storms by activating vagus nerve-induced inflammation [62]. However, the exact underlying mechanism remains unclear and should be elucidated in the future.

### 3.6. Allergic Rhinitis

Allergic rhinitis (AR) poses a significant global health concern, given its increasing incidence and substantial medical and socioeconomic impact in recent years [63]. The pathogenesis of allergic rhinitis is closely linked to the release of histamine, making H1 receptor antagonists the primary pharmacological intervention for treating this disease [64]. H1 and H2 receptors are both widely distributed in the nasal mucosa and capillary vessels [1]. A study conducted by Wood et el. demonstrated that the H1 receptor exerts the greatest effect on the alterations in nasal vascular permeability induced by topical histamine, while the H2 receptor exerts the greatest effect on nasal obstruction [20]. Therefore, the combination of H1 and H2 receptor antagonists can significantly reduce nasal airway resistance and increase nasal air flow in patients with allergic rhinitis [21].

### 3.7. Drug Hypersensitivity Reactions

In addition to being effective at treating the above histamine-mediated diseases, the combination of H1 and H2 receptor antagonists can also improve the clinical outcomes of patients suffering drug hypersensitivity reactions. Drug hypersensitivity reactions (DHRs) are defined as objectively reproducible signs or symptoms initiated by a drug at a dose usually tolerated by healthy individuals. The drugs that can induce DHRs include penicillin, nonsteroidal anti-inflammatory drugs (NSAIDs), chemotherapy drugs, narcotics, radiologic contrast dyes, etc. DHRs are usually not predictable and occur in susceptible individuals. Thus, they have become a serious threat to public health [65]. DHRs are mainly attributed to the release of histamine and triggering of mast cells, but the underlying mechanism remains unclear. The manifestations of DHRs include various signs and symptoms, such as cutaneous flushing, angioedema, cephalalgia, bronchospasm, hypotension, and even shock [66]. Despite the established efficacy of H1 receptor antagonists in treating histamine-induced DHRs, both the physiological rationale and case reports suggest that the combination of H1 and H2 receptor antagonists may yield superior outcomes compared to H1 receptor antagonists alone.

Chemotherapy drugs are one of the most common types of drugs to induce DHRs. Among them, carboplatin has been reported to induce severe DHRs, greatly limiting its clinical application for treating ovarian cancer [67]. Recently, several studies have shown the therapeutic effects of H1 and H2 receptor antagonists for carboplatin hypersensitivity reactions (CHRs) [68]. Navo M et al. conducted a retrospective chart review evaluating patients with all tumor types that received carboplatin and first confirmed that the combined use of H1 and H2 receptor antagonists could significantly reduce the risk of CHRs [68]. Another retrospective study evaluated the effects of the combination of H1 and H2 receptor antagonists in addition to dexamethasone as the standard pre-medication in women with ovarian cancer. The results showed that these patients were almost 50% less likely to develop CHRs than patients without premedication [69]. In addition, a case observation focusing on two children with pilocytic astrocytoma demonstrated that an H1 receptor antagonist or a combination of H1 and H2 receptor antagonists with prednisolone before carboplatin application can also reduce the incidence of CHRs, prevent head irradiation, and increase the chance of normal development of the nervous system, further illustrating the efficacy of antihistamine therapy for preventing CHRs [70].

Additionally, during general anesthesia, DHRs can be induced by narcotic drugs (opioids) and muscle relaxants, with the incidence of systemic reactions being 1–5% and life-threatening reactions being 0.1–0.5% [71]. A study conducted by Philbin et al. demonstrated that the combined use of diphenhydramine and cimetidine before morphine anesthesia can significantly prevent the occurrence of DHRs and help to obtain hemodynamic protection, which is superior to their administration alone [13]. Another double-blind study examined whether the combination of oral terfenadine and ranitidine before gynecologic surgery improved blood pressure reduction and skin flushing in patients after receiving tubocurarine and morphine [22]. Compared with those in the placebo group, the patients in the preoperative terfenadine and ranitidine groups had less hypotension and tachycardia but no significant decrease in cutaneous flushing immediately. These results suggest that the combination of H2 and first-generation H1 receptor antagonists can significantly attenuate cardiovascular changes, especially in high-risk patients and patients with previous allergic reactions to anesthesia. In addition, in some patients treated with chymopapain, H1 and H2 receptor antagonists might be useful prophylactics for immune-mediated reactions [72].

Each year, more than 60 million doses of iodinated contrast media are used worldwide, and iodinated contrast media has been associated with the potential occurrence of anaphylaxis shortly after administration [73]. To mitigate the risk of life-threatening anaphylactic reactions, premedication strategies involving the use of various drugs, including steroids, antihistamines, and others, either alone or in combination, are commonly employed [74]. In a prospective controlled trial conducted by Ring et al., the efficacy of clemastine in combination with cimetidine in preventing drug hypersensitivity reactions following the infusion of radiographic contrast media was identified, suggesting that the combined application of H1 and H2 antagonists might be useful in prophylaxis of radiographic contrast media-induced DHRs [14].

## 4. Potential Application of Combination Therapy Involving H1 and H2 Receptor Antagonists

Recently, emerging evidence has revealed that histamine plays indispensable roles in the development of central nervous system disorders, postexercise hypotension, arthritic diseases, and cardiac diseases. Therefore, the combined use of H1 and H2 receptor antagonists may exhibit remarkable potential in the management of these disorders and deserves additional attention in future clinical practice.

### 4.1. Central Nervous System Disorders

In the central nervous system, histamine serves as a neurotransmitter and plays a key role in several cerebral functions and physiological behavioral processes, such as the sleep–wake cycle, water and food intake, locomotion, memory, and learning [75]. A study conducted by Zarrindast et al. investigated the effects of intrahippocampal CA1 (intra-CA1) microinjection of histaminergic agents on anxiety-related behaviors in rats [16]. In this study, the rats were divided into eight groups that received either saline injections or different doses of pyrilamine, four of which also received histamine injections 5 min before the pyrilamine/ranitidine injections. The results showed that the effects of pyrilamine/ranitidine and histamine in the hippocampus are antagonistic, indicating an anxiogenic effect of the H1 and H2 receptor antagonists.

A cluster headache is a primary headache disorder that impacts approximately 0.1% of the global population [76]. It is widely acknowledged that episodes of cluster headaches involve dilation of external carotid vessels, which is believed to be the underlying cause of these headaches [77]. Histamine may play a critical role in the occurrence of vasodilatation and headaches; thus, the effects of histamine receptor antagonist treatment are worthy of attention [77]. J Cuypers et al. conducted a study involving the treatment of 13 patients with the H2 receptor antagonist cimetidine alone and/or in combination with the H1 receptor antagonist chlorpyramine [27]. The results suggested that the use of cimetidine alone was ineffective, while the combination of H1 and H2 receptor antagonists had satisfactory and prompt effects, especially for episodic cluster headaches [27].

Schizophrenia is a common and serious psychiatric disorder, and the current therapies for this disorder are unsatisfactory [78]. Recently, Katarina Meskanen et al. conducted a double-blind, placebo-controlled, parallel-group, randomized trial in patients with treatment-resistant schizophrenia treated with histamine receptor antagonists [33]. Thirty patients with schizophrenia were randomized to receive an oral antagonist or placebo in addition to their usual treatment regimen for 4 weeks. In the experimental group, the assessment of negative symptoms (SANS) score was reduced by 5.3 points, while the SANS score was virtually unchanged in the placebo group. Additionally, the positive and negative syndrome scale (PANSS) total score, the general subscore, and the CGI indicated greater changes in the experimental group than in the placebo group. These results suggest that histamine receptor antagonists may provide new alternatives for the treatment of schizophrenia.

### 4.2. Cardiac Diseases

Under normal physiological conditions, the heart harbors histamine, which exerts significant physiological effects on cardiac chronotropy and inotropy. In terms of cardiac diseases, numerous studies have elucidated the involvement of histamine in cardiac damage by interacting with various histamine receptors [79]. Both the H1R and H2R have been shown to elicit cardiac anaphylaxis and non-anaphylactic cardiac arrhythmias. Respectively, H1R is related to the coronary vascular reactivity and atherosclerosis, and the histamine-induced coronary spasm can be blocked by H1 receptor antagonists [80]. Meanwhile, the H2R exhibits adverse effects on cardiac remodeling and enhances heart failure through direct actions on cardiomyocytes, inducing hypertrophy, and on cardiac fibroblasts, causing fibrosis. Many clinical studies have demonstrated that the use of H2 receptor antagonists to block H2R improves the outcome of heart failure [81,82]. Additionally, short-term effects of H2R blockade on improving basic cardiovascular functions, such as decreasing contractility and cardiac output, have been investigated [83,84]. In contrast to H1R and H2R, the H3R may show a cardioprotective role by inhibiting norepinephrine (NE) release, which can reduce the occurrence of arrhythmias [85,86]. However, the longer-term effects of H3R are unclear. Moreover, the H3R has been shown to have anti-fibrotic properties chronically, which may contribute to its cardioprotective effects [86,87]. The link between the H4R and cardiac diseases is still unclear, making it a wide-open field for investigation and a potential target for the treatment of cardiac diseases.

Based on the above statements, the combination of H1 and H2 receptor antagonists may act as a potent therapy for the treatment of a variety of cardiac diseases, which deserves additional attention in the future.

### 4.3. Postexercise Hypotension

Postexercise hypotension is one of the most common causes of postexercise syncope and is a threat to endurance-trained and sedentary individuals [88]. However, the underlying mechanisms of postexercise hypotension have not been elucidated. Recent studies suggested that postexercise hypotension might be associated with H1 and H2 receptor mediated postexercise vasodilation [26]. In sedentary individuals, H1 receptors mediate the early portion (30 min after exercise) of postexercise skeletal muscle hyperemia, whereas H2 receptors mediate the latter portion (60 and 90 min after exercise) [26,89]. Subjects performed parallel exercise programs on two different experimental days and were randomized to receive treatment with combined H1 and H2 receptor antagonists or placebo. The results suggested that the administration of H1 and H2 receptor antagonists abolishes vasodilation after exercise and blunts postexercise hypotension in endurance exercise-trained and sedentary people.

Furthermore, Naylor et al. examined the effects of histamine H1 and H2 receptor blockade on blood pressure and hemodynamic responses via cardiac output, mean atrial pressure, aortic stiffness, and total vascular conductance at rest and during progressive cycling exercise in normal and high–normal blood pressure (BP) subjects [25]. Their findings indicated that the combination of H1 and H2 receptor antagonists could increase BP and hemodynamic responses during dynamic exercise in individuals with high–normal BP, with no significant change in diastolic blood pressure because the ability of H1 and H2 receptors to vasodilate is likely impaired [25].

## 5. Conclusions and Future Outlook

As a classic treatment strategy, the combination of H1 and H2 receptor antagonists has exhibited enhanced efficacy and stability in the treatment of diverse histamine-mediated disorders compared with single-drug therapies. Additionally, the combination strategy demonstrated significant potential in alleviating central nervous system disorders, arthritic diseases, cardiac diseases, and post-exercise hypotension. However, it is important to acknowledge that combination therapy is not without its drawbacks and adverse reactions. For example, H1R antagonists exhibit both inhibitory and agonistic effects on the central nervous system and can potentially affect daily life and work. These effects may be observed as drowsiness, reduced alertness, or restlessness, particularly with the use of first-generation H1 receptor antagonists. In addition, because of the effect of H2R antagonists on acid resistance, preoperative oral H1 and H2 receptor antagonists may reduce the production of gastric acid at the time of surgery, increasing the attendant risk of aspiration pneumonitis. Moreover, in view of the wide distribution of H1Rs and H2Rs in the heart, combination therapy may theoretically inhibit the function of the heart. Recently, several clinical studies have reported the incidence of arrhythmia caused by second-generation H1 receptor antagonists, such as terfenadine and astemizole.

In light of these issues, there are several ways in which we can mitigate and enhance the situation. First, the introduction of second-generation histamine receptor antagonists significantly decreases the occurrence of adverse reactions in the central nervous system. These antagonists exhibit a high degree of selectivity in binding to histamine receptors, making them the preferred choice for individuals engaged in hazardous occupations. However, individuals must refrain from operating machinery or engaging in potentially dangerous activities after consuming these medications to ensure their safety. Second, intravenous histamine receptor antagonists should be administered before extubating during surgery to mitigate the likelihood of aspiration pneumonitis rather than via oral administration. Finally, caution should be exercised when employing combination therapy in patients with cardiac insufficiency.

In addition to the conventional H1 and H2 receptor antagonists, there have been emerging studies on H3 and H4 receptor antagonists in recent years. The H3R is predominately located presynaptically on neurons and acts as an important neurotransmitter or neuromodulator [90]. Recently, a variety of H3 receptor antagonists have been used to treat neurological disorders, such as epilepsy, parkinsonism, and narcolepsy, and in the process of neurological regeneration [91]. For example, pitolisant, the first in-class antagonist of the H3 receptor, has been used in the clinical treatment of narcolepsy and the treatment of neuropsychiatric disorders, such as schizophrenia and epilepsy, in some ongoing clinical studies [92,93]. The H4R is the least studied of the four identified histamine receptors and it shares the highest sequence similarity with the H3R. H4Rs are widely distributed in several tissues and exert various effects on the production of cytokine and chemokine in mast cells, eosinophils, dendritic cells, and T cells. Thus, H4 receptor antagonists may act as novel drugs for the treatment of several refractory diseases. Notably, the combination therapies among H1, H2, H3, and H4 receptor antagonists may bring new directions for the treatment of histamine-mediated diseases.

To summarize, the combination of H1 and H2 receptor antagonists has traditionally been regarded as a classic therapeutic approach for a variety of histamine-mediated disorders and has exhibited remarkable superiority compared with single-drug therapies. Furthermore, although the exact mechanism is still unclear, combination therapy may also present a novel avenue for addressing anxiety disorders, tumors, postexercise hypotension, and arthritic diseases, which deserves additional attention in future clinical practice.

## Figures and Tables

**Table 1:** The H1R and H2R antagonists that are currently clinically used.

Types	Drugs	Structure	Refs.
H1R antagonists			
First-generation	Diphenhydramine	Ethanolamine	[11,12,13]
	Clemastine	Ethanolamine	[14]
	Chlorpheniramine	Hydrocarbon amine	[15]
	Pyrilamine/Mepyramine	Ethylenediamine	[16,17]
	Cyproheptadine	Piperidine	[18]
Second-generation	Cetirizine	Piperazine	[19,20,21]
	Terfenadine	Piperidine	[22,23,24]
Third-generation	Fexofenadine	Piperidine	[25,26]
H2R antagonists			
First-generation	Cimetidine	Imidazole	[11,13,14,15,27]
Second-generation	Ranitidine	Furan	[12,16,17,22,23]
	Lafutidine/loxtidine	Furan	[28]
Third-generation	Famotidine	Thiazole	[19]
Fourth generation	Roxatidine	Piperidine toluene	[24]

## Data Availability

No new data were created or analyzed in this study. Data sharing is not applicable to this article.

## References

[1] Panula P., Chazot P.L., Cowart M., Gutzmer R., Leurs R., Liu W.L., Stark H., Thurmond R.L., Haas H.L. (2015). International Union of Basic and Clinical Pharmacology. XCVIII. Histamine Receptors. Pharmacol. Rev..

[2] Hill S.J. (1990). Distribution, properties, and functional characteristics of three classes of histamine receptor. Pharmacol. Rev..

[3] Sander L.E., Lorentz A., Sellge G., Coëffier M., Neipp M., Veres T., Frieling T., Meier P.N., Manns M.P., Bischoff S.C. (2006). Selective expression of histamine receptors H1R, H2R, and H4R, but not H3R, in the human intestinal tract. Gut.

[4] Zhang M., Thurmond R.L., Dunford P.J. (2007). The histamine H_4_ receptor: A novel modulator of inflammatory and immune disorders. Pharmacol. Ther..

[5] Simons F.E., Simons K.J. (2011). Histamine and H_1_-antihistamines: Celebrating a century of progress. J. Allergy Clin. Immunol..

[6] Thangam E.B., Jemima E.A., Singh H., Baig M.S., Khan M., Mathias C.B., Church M.K., Saluja R. (2018). The Role of Histamine and Histamine Receptors in Mast Cell-Mediated Allergy and Inflammation: The Hunt for New Therapeutic Targets. Front. Immunol..

[7] Zuberbier T., Peter J., Staubach P., Chularojanamontri L., Kulthanan K. (2023). Potential Therapeutic Approaches for Chronic Urticaria: Beyond H_1_-Antihistamines and Biologics. J. Allergy Clin. Immunol. Pract..

[8] Zhou S., Huang G. (2020). Synthesis of anti-allergic drugs. RSC Adv..

[9] He A., Feldman S.R., Fleischer A.B. (2018). An assessment of the use of antihistamines in the management of atopic dermatitis. J. Am. Acad. Dermatol..

[10] van Pinxteren B., Numans M.E., Bonis P.A., Lau J. (2006). Short-term treatment with proton pump inhibitors, H_2_-receptor antagonists and prokinetics for gastro-oesophageal reflux disease-like symptoms and endoscopy negative reflux disease. Cochrane Database Syst. Rev..

[11] Runge J.W., Martinez J.C., Caravati E.M., Williamson S.G., Hartsell S.C. (1992). Histamine antagonists in the treatment of acute allergic reactions. Ann. Emerg. Med..

[12] Lin R.Y., Curry A., Pesola G.R., Knight R.J., Lee H.S., Bakalchuk L., Tenenbaum C., Westfal R.E. (2000). Improved outcomes in patients with acute allergic syndromes who are treated with combined H_1_ and H_2_ antagonists. Ann. Emerg. Med..

[13] Philbin D.M., Moss J., Akins C.W., Rosow C.E., Kono K., Schneider R.C., VerLee T.R., Savarese J.J. (1981). The use of H_1_ and H_2_ histamine antagonists with morphine anesthesia: A double-blind study. Anesthesiology.

[14] Ring J., Rothenberger K.H., Clauss W. (1985). Prevention of anaphylactoid reactions after radiographic contrast media infusion by combined histamine H_1_- and H_2_-receptor antagonists: Results of a prospective controlled trial. Int. Arch. Allergy Immunol..

[15] Diller G., Orfanos C.E. (1983). Management of idiopathic urticaria with H_1_ + H_2_ antagonists. A crossover double blind long-term study. Z. Hautkrankh..

[16] Zarrindast M.R., Torabi M., Rostami P., Fazli-Tabaei S. (2006). The effects of histaminergic agents in the dorsal hippocampus of rats in the elevated plus-maze test of anxiety. Pharmacol. Biochem. Behav..

[17] Kennedy L., Hargrove L., Demieville J., Karstens W., Jones H., DeMorrow S., Meng F., Invernizzi P., Bernuzzi F., Alpini G. (2018). Blocking H_1_/H_2_ histamine receptors inhibits damage/fibrosis in Mdr2(−/−) mice and human cholangiocarcinoma tumorigenesis. Hepatology.

[18] Gasior-Chrzan B., Falk E.S. (1992). Systemic mastocytosis treated with histamine H_1_ and H_2_ receptor antagonists. Dermatology.

[19] Hogan Ii R.B., Hogan Iii R.B., Cannon T., Rappai M., Studdard J., Paul D., Dooley T.P. (2020). Dual-histamine receptor blockade with cetirizine—Famotidine reduces pulmonary symptoms in COVID-19 patients. Pulm. Pharmacol. Ther..

[20] Wood-Baker R., Lau L., Howarth P.H. (1996). Histamine and the nasal vasculature: The influence of H_1_ and H_2_-histamine receptor antagonism. Clin. Otolaryngol. Allied Sci..

[21] Wang D., Clement P., Smitz J. (1996). Effect of H_1_ and H_2_ antagonists on nasal symptoms and mediator release in atopic patients after nasal allergen challenge during the pollen season. Acta Oto-Laryngol..

[22] Treuren B.C., Galletly D.C., Robinson B.J., Short T.G., Ure R.W. (1993). The influence of the H_1_ and H_2_ receptor antagonists, terfenadine and ranitidine on the hypotensive and gastric pH effects of the histamine releasing drugs, morphine and tubocurarine. Anaesthesia.

[23] Paul E., Bödeker R.H. (1986). Treatment of chronic urticaria with terfenadine and ranitidine. A randomized double-blind study in 45 patients. Eur. J. Clin. Pharmacol..

[24] Modlin I.M., Zhu Z., Tang L.H., Kidd M., Lawton G.P., Miu K., Powers R.E., Goldenring J.R., Pasikhov D., Soroka C.J. (1996). Evidence for a regulatory role for histamine in gastric enterochromaffin-like cell proliferation induced by hypergastrinemia. Digestion.

[25] Naylor A., Shariffi B., Gillum T.L., William B., Sullivan S., Kim J.K. (2020). Effects of combined histamine H_1_ and H_2_ receptor blockade on hemodynamic responses to dynamic exercise in males with high-normal blood pressure. Appl. Physiol. Nutr. Metab..

[26] McCord J.L., Halliwill J.R. (2006). H_1_ and H_2_ receptors mediate postexercise hyperemia in sedentary and endurance exercise-trained men and women. J. Appl. Physiol..

[27] Cuypers J., Altenkirch H., Bunge S. (1979). Therapy of cluster headache with histamine H_1_ and H_2_ receptor antagonists. Eur. Neurol..

[28] Ogawa Y., Ichinokawa Y., Hiruma M., Machida Y., Funakushi N., Sadamasa H., Hiruma M. (2013). Retrospective cohort study on combination therapy with the histamine H_2_-receptor antagonist lafutidine for antihistamine-resistant chronic urticaria. J. Dermatol. Treat..

[29] Ring J., Behrendt H. (1990). H_1_- and H_2_-antagonists in allergic and pseudoallergic diseases. Clin. Exp. Allergy.

[30] Curto-Barredo L., Giménez-Arnau A.M. (2019). Treatment of chronic spontaneous urticaria with an inadequate response to H_1_-antihistamine. Ital. J. Dermatol. Venereol..

[31] Castells M., Butterfield J. (2019). Mast Cell Activation Syndrome and Mastocytosis: Initial Treatment Options and Long-Term Management. J. Allergy Clin. Immunol. Pract..

[32] ALMuhizi F., De Las Vecillas Sanchez L., Gilbert L., Copaescu A.M., Isabwe G.A.C. (2022). Premedication Protocols to Prevent Hypersensitivity Reactions to Chemotherapy: A Literature Review. Clin. Rev. Allergy Immunol..

[33] Meskanen K., Ekelund H., Laitinen J., Neuvonen P.J., Haukka J., Panula P., Ekelund J. (2013). A randomized clinical trial of histamine 2 receptor antagonism in treatment-resistant schizophrenia. J. Clin. Psychopharmacol..

[34] Marzaioli V., McMorrow J.P., Angerer H., Gilmore A., Crean D., Zocco D., Rooney P., Veale D., Fearon U., Gogarty M. (2012). Histamine contributes to increased RANKL to osteoprotegerin ratio through altered nuclear receptor 4A activity in human chondrocytes. Arthritis Rheum..

[35] Lin R.Y., Schwartz L.B., Curry A., Pesola G.R., Knight R.J., Lee H.S., Bakalchuk L., Tenenbaum C., Westfal R.E. (2000). Histamine and tryptase levels in patients with acute allergic reactions: An emergency department-based study. J. Allergy Clin. Immunol..

[36] Eraky A.M., Wright A., McDonald D. (2023). Pseudo-Allergies in the Emergency Department: A Common Misdiagnosis of Hypersensitivity Type 1 Allergic Reaction. Cureus.

[37] Cmorej P.C., Nesvadba M., Babeľa R., Slowik O., Didič R. (2017). Management of acute anaphylaxis in clinical practice in the context of the guidelines. Epidemiol. Mikrobiol. Imunol..

[38] Lang D.M. (2022). Chronic Urticaria. N. Engl. J. Med..

[39] O’Donnell B.F. (2014). Urticaria: Impact on quality of life and economic cost. Immunol. Allergy Clin. N. Am..

[40] Zuberbier T., Abdul Latiff A.H., Abuzakouk M., Aquilina S., Asero R., Baker D., Ballmer-Weber B., Bangert C., Ben-Shoshan M., Bernstein J.A. (2022). The international EAACI/GA^2^LEN/EuroGuiDerm/APAAACI guideline for the definition, classification, diagnosis, and management of urticaria. Allergy.

[41] Lee N., Lee J.D., Lee H.Y., Kang D.R., Ye Y.M. (2017). Epidemiology of Chronic Urticaria in Korea Using the Korean Health Insurance Database, 2010–2014. Allergy Asthma Immunol. Res..

[42] Wang Y.M., Du L., Zhu Y.J. (2017). Evidence-based therapies of Chinese medicine for chronic urticaria: Where do we stand and where are we going?. Chin. J. Integr. Med..

[43] Bernstein J.A., Lang D.M., Khan D.A., Craig T., Dreyfus D., Hsieh F., Sheikh J., Weldon D., Zuraw B., Bernstein D.I. (2014). The diagnosis and management of acute and chronic urticaria: 2014 update. J. Allergy Clin. Immunol..

[44] Phanuphak P., Schocket A., Kohler P.F. (1978). Treatment of chronic idiopathic urticaria with combined H_1_ and H_2_ blockers. Clin. Exp. Allergy.

[45] Singh G. (1984). H_2_ blockers in chronic urticaria. Int. J. Dermatol..

[46] Derakhshani A., Vahidian F., Alihasanzadeh M., Mokhtarzadeh A., Lotfi Nezhad P., Baradaran B. (2019). Mast cells: A double-edged sword in cancer. Immunol. Lett..

[47] Bonato G., Cristoferi L., Strazzabosco M., Fabris L. (2015). Malignancies in Primary Sclerosing Cholangitis—A Continuing Threat. Dig. Dis..

[48] Yimam K.K., Bowlus C.L. (2014). Diagnosis and classification of primary sclerosing cholangitis. Autoimmun. Rev..

[49] Jones H., Hargrove L., Kennedy L., Meng F., Graf-Eaton A., Owens J., Alpini G., Johnson C., Bernuzzi F., Demieville J. (2016). Inhibition of mast cell-secreted histamine decreases biliary proliferation and fibrosis in primary sclerosing cholangitis Mdr2(−/−) mice. Hepatology.

[50] Johnson C., Huynh V., Hargrove L., Kennedy L., Graf-Eaton A., Owens J., Trzeciakowski J.P., Hodges K., DeMorrow S., Han Y. (2016). Inhibition of Mast Cell-Derived Histamine Decreases Human Cholangiocarcinoma Growth and Differentiation via c-Kit/Stem Cell Factor-Dependent Signaling. Am. J. Pathol..

[51] Francis H., DeMorrow S., Venter J., Onori P., White M., Gaudio E., Francis T., Greene J.F., Tran S., Meininger C.J. (2012). Inhibition of histidine decarboxylase ablates the autocrine tumorigenic effects of histamine in human cholangiocarcinoma. Gut.

[52] Francis H.L., Demorrow S., Franchitto A., Venter J.K., Mancinelli R.A., White M.A., Meng F., Ueno Y., Carpino G., Renzi A. (2012). Histamine stimulates the proliferation of small and large cholangiocytes by activation of both IP3/Ca2+ and cAMP-dependent signaling mechanisms. Lab. Investig..

[53] Shi Z., Fultz R.S., Engevik M.A., Gao C., Hall A., Major A., Mori-Akiyama Y., Versalovic J. (2019). Distinct roles of histamine H_1_- and H_2_-receptor signaling pathways in inflammation-associated colonic tumorigenesis. Am. J. Physiol.-Gastrointest. Liver Physiol..

[54] Valent P., Akin C., Sperr W.R., Horny H.P., Arock M., Metcalfe D.D., Galli S.J. (2023). New Insights into the Pathogenesis of Mastocytosis: Emerging Concepts in Diagnosis and Therapy. Annu. Rev. Pathol..

[55] Zanotti R., Tanasi I., Crosera L., Bonifacio M., Schena D., Orsolini G., Mastropaolo F., Tebaldi M., Olivieri E., Bonadonna P. (2021). Systemic Mastocytosis: Multidisciplinary Approach. Mediterr. J. Hematol. Infect. Dis..

[56] Worobec A.S. (2000). Treatment of systemic mast cell disorders. Hematol. Oncol. Clin. N. Am..

[57] Guan W.J., Ni Z.Y., Hu Y., Liang W.H., Ou C.Q., He J.X., Liu L., Shan H., Lei C.L., Hui D.S.C. (2020). Clinical Characteristics of Coronavirus Disease 2019 in China. N. Engl. J. Med..

[58] Wu C., Liu Y., Yang Y., Zhang P., Zhong W., Wang Y., Wang Q., Xu Y., Li M., Li X. (2020). Analysis of therapeutic targets for SARS-CoV-2 and discovery of potential drugs by computational methods. Acta Pharm. Sin. B.

[59] Shaffer L. (2020). 15 drugs being tested to treat COVID-19 and how they would work. Nat. Med..

[60] Morán Blanco J.I., Alvarenga Bonilla J.A., Homma S., Suzuki K., Fremont-Smith P., Villar Gómez de Las Heras K. (2021). Antihistamines and azithromycin as a treatment for COVID-19 on primary health care—A retrospective observational study in elderly patients. Pulm. Pharmacol. Ther..

[61] Pinto M.D., Lambert N., Downs C.A., Abrahim H., Hughes T.D., Rahmani A.M., Burton C.W., Chakraborty R. (2022). Antihistamines for Postacute Sequelae of SARS-CoV-2 Infection. J. Nurse Pract..

[62] Yang H., George S.J., Thompson D.A., Silverman H.A., Tsaava T., Tynan A., Pavlov V.A., Chang E.H., Andersson U., Brines M. (2022). Famotidine activates the vagus nerve inflammatory reflex to attenuate cytokine storm. Mol. Med..

[63] Zhang Y., Lan F., Zhang L. (2022). Update on pathomechanisms and treatments in allergic rhinitis. Allergy.

[64] Ziering R.W., Klein G.L. (1992). Allergic rhinitis. Measures to control the misery. Postgrad. Med..

[65] Demoly P., Adkinson N.F., Brockow K., Castells M., Chiriac A.M., Greenberger P.A., Khan D.A., Lang D.M., Park H.S., Pichler W. (2014). International Consensus on drug allergy. Allergy.

[66] Wang Z., Wang D., Sui Y., Cui H., Yu Y. (2012). Experimental study on anaphylaxis of qingkailing injection and its components on Beagle dogs. J. Tradit. Chin. Med..

[67] Colombo N., Peiretti M., Parma G., Lapresa M., Mancari R., Carinelli S., Sessa C., Castiglione M. (2010). Newly diagnosed and relapsed epithelial ovarian carcinoma: ESMO Clinical Practice Guidelines for diagnosis, treatment and follow-up. Ann. Oncol..

[68] Navo M., Kunthur A., Badell M.L., Coffer L.W., Markman M., Brown J., Smith J.A. (2006). Evaluation of the incidence of carboplatin hypersensitivity reactions in cancer patients. Gynecol. Oncol..

[69] Mach C.M., Lapp E.A., Weddle K.J., Hunter R.J., Burns K.A., Parker C., Brown J., Smith J.A. (2016). Adjunct Histamine Blockers as Premedications to Prevent Carboplatin Hypersensitivity Reactions. Pharmacotherapy.

[70] Broome C.B., Schiff R.I., Friedman H.S. (1996). Successful desensitization to carboplatin in patients with systemic hypersensitivity reactions. Med. Pediatr. Oncol..

[71] Stoelting R.K., Gibbs P.S. (1973). Hemodynamic effects of morphine and morphine-nitrous oxide in valvular heart disease and coronary-artery disease. Anesthesiology.

[72] Javid M.J., Nordby E.J., Ford L.T., Hejna W.J., Whisler W.W., Burton C., Millett D.K., Wiltse L.L., Widell E.H., Boyd R.J. (1983). Safety and efficacy of chymopapain (Chymodiactin) in herniated nucleus pulposus with sciatica. Results of a randomized, double-blind study. JAMA.

[73] Boehm I., Nairz K., Morelli J., Silva Hasembank Keller P., Heverhagen J.T. (2017). General anaesthesia for patients with a history of a contrast medium-induced anaphylaxis: A useful prophylaxis?. Br. J. Radiol..

[74] Bilò M.B., Martini M., Tontini C., Mohamed O.E., Krishna M.T. (2019). Idiopathic anaphylaxis. Clin. Exp. Allergy.

[75] Onodera K., Miyazaki S., Imaizumi M., Stark H., Schunack W. (1998). Improvement by FUB 181, a novel histamine H3-receptor antagonist, of learning and memory in the elevated plus-maze test in mice. Naunyn-Schmiedeberg's Arch. Pharmacol..

[76] Russell M.B. (2004). Epidemiology and genetics of cluster headache. Lancet Neurol..

[77] Worm J., Falkenberg K., Olesen J. (2019). Histamine and migraine revisited: Mechanisms and possible drug targets. J. Headache Pain.

[78] Liberman R.P., Kopelowicz A. (2005). Recovery from schizophrenia: A concept in search of research. Psychiatr. Serv..

[79] Gergs U., Kirchhefer U., Bergmann F., Künstler B., Mißlinger N., Au B., Mahnkopf M., Wache H., Neumann J. (2020). Characterization of Stressed Transgenic Mice Overexpressing H_2_-Histamine Receptors in the Heart. J. Pharmacol. Exp. Ther..

[80] Shimokawa H., Tomoike H., Nabeyama S., Yamamoto H., Nakamura M. (1985). Histamine-induced spasm not significantly modulated by prostanoids in a swine model of coronary artery spasm. J. Am. Coll. Cardiol..

[81] Kim J., Ogai A., Nakatani S., Hashimura K., Kanzaki H., Komamura K., Asakura M., Asanuma H., Kitamura S., Tomoike H. (2006). Impact of blockade of histamine H_2_ receptors on chronic heart failure revealed by retrospective and prospective randomized studies. J. Am. Coll. Cardiol..

[82] He G.H., Xu G.L., Cai W.K., Zhang J. (2016). Is Histamine H_2_ Receptor a Real Promising Target for Prevention or Treatment of Heart Failure?. J. Am. Coll. Cardiol..

[83] Hinrichsen H., Halabi A., Kirch W. (1990). Hemodynamic effects of different H_2_-receptor antagonists. Clin. Pharmacol. Ther..

[84] Halabi A., Nokhodian A., Kirch W. (1992). Haemodynamic effects of roxatidine, an H_2_-receptor antagonist. Clin. Investig..

[85] Imamura M., Seyedi N., Lander H.M., Levi R. (1995). Functional identification of histamine H_3_-receptors in the human heart. Circ. Res..

[86] Levi R., Seyedi N., Schaefer U., Estephan R., Mackins C.J., Tyler E., Silver R.B. (2007). Histamine H_3_-receptor signaling in cardiac sympathetic nerves: Identification of a novel MAPK-PLA2-COX-PGE2-EP3R pathway. Biochem. Pharmacol..

[87] Piera L., Olczak S., Kun T., Galdyszynska M., Ciosek J., Szymanski J., Drobnik J. (2019). Disruption of histamine/H_3_ receptor signal reduces collagen deposition in cultures scar myofibroblasts. J. Physiol. Pharmacol..

[88] Lei T.H., Fujii N., Zhang X., Wang F., Mündel T., Wang I.L., Chen Y.M., Nishiyasu T., Amano T., Dobashi K. (2023). The effects of high-intensity exercise training and detraining with and without active recovery on postexercise hypotension in young men. Physiol. Rep..

[89] Lockwood J.M., Wilkins B.W., Halliwill J.R. (2005). H_1_ receptor-mediated vasodilatation contributes to postexercise hypotension. J. Physiol..

[90] Nieto-Alamilla G., Márquez-Gómez R., García-Gálvez A.M., Morales-Figueroa G.E., Arias-Montaño J.A. (2016). The Histamine H3 Receptor: Structure, Pharmacology, and Function. Mol. Pharmacol..

[91] Harwell V., Fasinu P.S. (2020). Pitolisant and Other Histamine-3 Receptor Antagonists-An Update on Therapeutic Potentials and Clinical Prospects. Medicines.

[92] Lamb Y.N. (2020). Pitolisant: A Review in Narcolepsy with or without Cataplexy. CNS Drugs.

[93] Zaccara G., Bartolini E., Tramacere L., Lattanzi S. (2021). Drugs for patients with epilepsy and excessive daytime sleepiness. Epilepsy Behav..

